# Monitoring Performance Degradation of Cerebellar Functions Using Computational Neuroscience Methods: Implications on Neurological Diseases

**DOI:** 10.1371/journal.pone.0045581

**Published:** 2012-09-20

**Authors:** Robert A. Nawrocki, Majid Shaalan, Sean E. Shaheen, Nancy M. Lorenzon

**Affiliations:** 1 Department of Computer Engineering, University of Denver, Denver, Colorado, United States of America; 2 Department of Computer Science and Engineering, University of Colorado Denver, Denver, Colorado, United States of America; 3 Department of Physics and Astronomy, University of Denver, Denver, Colorado, United States of America; 4 Department of Biological Sciences, University of Denver, Denver, Colorado, United States of America; Charité University Medicine Berlin, Germany

## Abstract

Neurodegeneration is a major cause of human disease. Within the cerebellum, neuronal degeneration and/or dysfunction has been associated with many diseases, including several forms of cerebellar ataxia, since normal cerebellar function is paramount for proper motor coordination, balance, and motor learning. The cerebellum represents a well-established neural circuit. Determining the effects of neuronal loss is of great importance for understanding the fundamental workings of the cerebellum and disease-associated dysfunctions. This paper presents computational modeling of cerebellar function in relation to neurodegeneration either affecting a specific cerebellar cell type, such as granule cells or Purkinje cells, or more generally affecting cerebellar cells and the implications on effects in relation to performance degradation throughout the progression of cell death. The results of the models show that the overall number of cells, as a percentage of the total cell number in the model, of a particular type and, primarily, their proximity to the circuit output, and not the neuronal convergence due to the relative number of cells of a particular type, is the main indicator of the gravity of the functional deficit caused by the degradation of that cell type. Specifically, the greater the percentage loss of neurons of a specific type and the closer proximity of those cells to the deep cerebellar neurons, the greater the deficit caused by the neuronal cell loss. These findings contribute to the understanding of the functional consequences of neurodegeneration and the functional importance of specific connectivity within a neuronal circuit.

## Introduction

Cognition and other mental processes are manifestations of neuronal computation, and as such they are acutely amenable to computational analysis [1], [2]. A number of research groups have conducted computational analyses of brain structures with varying degrees of cellular detail or function outcome. O'Reilly *et al*. [3], [4], [5], [6], [7] have modeled several brain regions, including hippocampus, neocortex, and basal ganglia, as well as a number of cognitive processes. Their tool of analysis is a software program called Emergent, which is unique in the world of computational neuroscience through its use of LEABRA [3], [8], [9], a biologically realistic learning algorithm. Howell *et al*. [10] conducted extensive studies of the cerebellum using PGENESIS, a simulation environment developed at Caltech that allows for analysis at many layers including subcellular processes, individual neurons, networks of neurons, and neuronal systems. Medina and Mauk [11], [12] conducted experiments aiming at modeling cerebellum and its cognitive functions by building a model reflecting the known cellular and synaptic components and a training pattern based on rabbit eyelid conditioning. Rossant *et al.*, using the Brian neural simulator [13], have approximated the electrophysiological recordings of neural responses to somatically injected currents of cortical neurons [14].

However, few computational neuroscientific studies relating to neural damage or neurodegeneration have been published. In one of the earlier studies, Devlin *et al.* modeled both localized and wide spread brain damage with the aim of understanding the degeneration associated with the progression of Alzheimer’s disease [15]. Their model was a high-level, semantic one consisting of two layers, labeled Semantics and Phonology, each with their own hidden layer called Semantic Clean-Up and Phonological Clean-up, respectively. They verified that their model produced results, vis-a-vis the degree of impairment over the course of semantic deterioration, that were consistent with the existing patient data. In another study, a mathematical model, based on plasticity instantiated by an activity-dependent rewiring rule, was constructed to study the interplay between synaptogenesis, neuronal death, and neurogenesis on the resulting pattern of neuronal connectivity [16]. The authors found that activity-dependent plasticity yields a robust network, while target deletion of central nodes leads to a drop in global efficiency. In yet another investigation, Alstott *et al.*, have constructed a computational model to investigate the functional consequences of lesions placed in different regions of the cerebral cortex [17]. They found that the magnitude of the impairment depends on the lesion location: lesions along the cortical midline result in large and widely distributed changes in functional connectivity, while the effects of lesions of primary sensory or motor regions remain more localized. Such sensitivity to the location within a sub-network of the brain is precisely the focus of the present study.

The model implemented here represents a real neuronal circuit, the cerebellum, as described by Ito [18] and Medina and Mauk [11], [12]. Our model represents a single microzone [18], explained in more detail in the *Cerebellum Architecture* section, which is a building block of the system being modeled. The research presented in this paper concentrates on modeling the cerebellum with the emphasis on cellular organization, connectivity, and neural projection as well as a training task. The computational model incorporates established neuronal components and features such as relative numbers of individual cell types, their spatial and influential relationship to one another, as well as input stimuli used during training. The model was used to study the functional effects of different patterns of neurodegeneration within the cerebellum with the primary goal of understanding the importance of cellular organization on the loss of skills during the progression of a disease.

Certain diseases have a well-defined neurologic target primarily affecting an individual cell type, while other diseases more indiscriminately or generally affect brain regions. For instance, the autosomal dominant episodic ataxias and spinocerebellar ataxias (SCAs) are a group of human diseases that mainly affect the Purkinje cells of the cerebellum [19]. In contrast, Creutzfeldt-Jacob disease (CJD) in humans is a typical prion diseases that less discriminately affects the cerebellum; however, the neurodegeneration is primarily of granule cells [20]. In addition, neurovascular or traumatic insults to the cerebellum would affect cells by location of the insult and not necessarily in a cell-type specific manner. Cerebellar neurodegeneration is even observed after insult to more distant brain regions (e.g., multiple sclerosis, brain trauma, and stroke); thus, the resultant cerebellar cell death is considered ”remote cell death” [21]. The relative ease of modeling certain neurological diseases comes from the aforementioned fact that the pattern of cell loss is fairly well documented and facilitates modeling of those diseases by “loss” of cerebellar neurons.

## Materials and Methods

This research was conducted using the Emergent™ software platform. Emergent was originally developed at Carnegie Mellon University circa 1995 under the name of PDP++. Currently, the software is being maintained and developed by the O'Reilly group at University of Colorado at Boulder [9]. The software was developed for the purposes of modeling neural network architectures with the ability to include biologically-inspired neural and cognitive functions. While it provides conventional learning algorithms, such as backpropagation or Kohonen Self Organizing Map (KSOM), for biologically plausible analysis it provides a unique learning algorithm with LEABRA, or Local Error-driven, and Associative Biologically Realistic Algorithm [3], [8], [9]. LEABRA is based on a balance between Hebbian and error-driven learning with a point-neuron activation function. With LEABRA, Emergent provides for biologically realistic simulations while allowing the speciation of the input pattern in a convenient, numeric form (for example “01” could be used for a cat, “10” used for a dog, “11” used for both cat and a dog, and “00” used for neither).

### Computational Models

There are two general approaches when constructing a simulation model; the top-down, and the bottom-up approach [22]. In a top-down approach, an overview of the system is formulated, concentrating on the relationship between individual “black boxes” that perform specific functions. According to Medina and Mauk, the construction of this type of model is characterized by first devising a real-time computational model that describes the behavioral and physiological evidence followed by devising an implementation that aligns the features of the model with the neural circuit involved [11]. A model constructed according to this approach benefits from the fact that the model is constructed specifically to produce output in accordance to found neurological evidence. However, the neurological plausibility of such a model is often questioned.

A bottom-up model is based on smallest subsystems that are connected together to form a larger system. This approach starts from known properties of individual neurons and their interconnections [22]. Such simulations are built by utilizing empirically determined anatomical and physiological constraints. Because neurons are too complex in their structure, variety, and operation, only a fraction of the known properties are included in the models, typically including numerical ratios, divergence and convergence ratios, synaptic strengths, connection geometry, and inhibitory and excitatory connection types [11]. An often cited drawback of this approach is the lack of biological plausibility stemming from the general lack of sufficiently detailed neuronal parameters being included as well as often lumping separate cell types into monolithic blocks. However, some processes being intrinsically bottom-up processes, such as fast reaction of visual identification, are better characterized using this modeling paradigm as they rely on primarily on sensory information [23].

The model constructed for this study was formulated according to the bottom-up approach. Undoubtedly, our model is not entirely consistent to that observed in the real biological system due to omission of some neural parameters. However, following the guidelines outlined in [11] as well as neuronal details presented in [4], [5], [11], [18], further validated by the ability to successfully simulate the Hull’s test (discussed later in text), we believe that the biological plausibility of the simulation has been substantiated.

It should be noted that, in vivo neurons within the brain are connected in a full, three-dimensional space. However, in a computer simulation such a space is purely abstract. For the purposes of visualization, the model is presented as a two-dimensional environment.

### Single Neuron Model

LEABRA implements a balance between Hebbian and error-driven learning on top of a biologically-based point-neuron activation function with inhibitory competition dynamics, either via inhibitory interneurons or a fast k-Winners-Take-All approximation [9]. The point-neuron activation function, with both discrete spiking and continuous rate-code output, models the electrophysiological properties of real neurons while simplifying their geometries to single discrete units. The membrane potential *V_m_* is updated as a function of ionic conductances *g* (*g_c_* is a time-varying component computed as a function of the dynamic state of the network, while 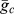 is a constant that controls the relative influence of the different conductances) with reversal (driving) potentials *E* and a calcium kinetics time constant *τ*:
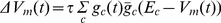
(1)


Hebbian learning is performed using a Conditional Principal Components Analysis (CPCA) algorithm with a correction factor for sparse expected activity levels [3]. The error-driven learning is achieved with GeneRec; the output is computed in two phases – an expectation phase where the network's actual output is produced and an outcome phase where the target output is experienced – as a difference of a pre- and postsynaptic activation product across these two phases. Hebbian weights are adjusted according to the following formula.
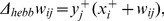
(2)


while error-driven learning uses the following equation.
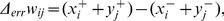
(3)where *x_i_* is the input of neuron *i*, *y_j_* is the output of neuron *j*, and *w_ij_* is the connection weight between neurons *i* and *j*. The “+” and “– superscripts refer to plus and minus phases of the GeneRec algorithm.

Biological neurons encode information via frequency modulation and as such can be directly simulated using spiking neural networks. However, simulations commonly operate using amplitude modulation (non-spiking neural networks) to simplify the computational complexity without the loss of functionality or generalizeability. The validity of using amplitude modulation as a simplified mapping of frequency modulation is well established (see for instance Aisa in [8]) and will not be addressed here.

### Cerebellum Architecture

**Figure 1:**
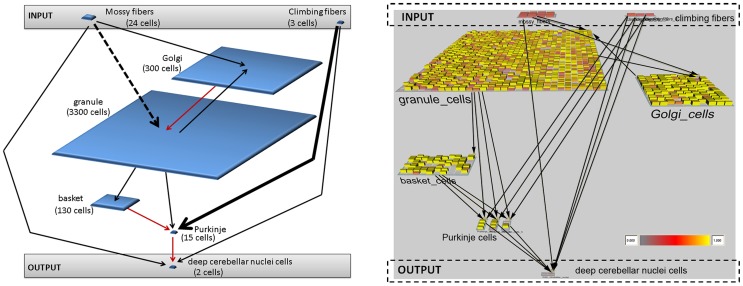
Organization of cells and connectivity of the model. (**a**) Number of cells is indicated in parenthesis. Arrows indicate connections between individual cell types. The thick arrow (between climbing fibers and Purkinje cells) represents the strongest connection (about 26,000 the strength of the rest of connections). The thick, dashed arrow (between mossy fibers and granule cells) represents a connection that is about four times as strong as the rest of the connections. Black arrows indicate excitatory connections, while red indicate inhibitory connections (between Golgi cells and granule cells, granule cells and basket cells, and basket cells and Purkinje cells). (**b**) Snapshot of the simulation with the emphasis on the cerebellar topology. Different cell types, grouped into blocks, show separate cells, facilitated by the overall number of cells of a specific type. The arrows between individual cell types indicate the neuronal connectivity. The colors of individual blocks within individual cell type indicate the strength of a neuronal output of a specific cell with the color range being indicated in the figure; yellow block indicates strongest relative numerical output, while orange and red blocks indicate decreasingly weaker outputs, respectively.

The cerebellum is known to contain over 50% of the neurons of the brain even though its size comprises only about 10%. This brain region is a good candidate for computational simulation since cerebellar architecture is well mapped with distinct cell types and connections. In addition, the cerebellar circuitry can be divided into relatively self-contained microzones [18] that are formed by a number of cell groups. In the current study, the model represents an individual microzone. Our microzone was defined as containing inputs from mossy fibers and climbing fibers, intrinsic cell types of granule cells, Golgi cells, basket cells, and Purkinje cells, and an output of deep cerebellar neurons. In this present study, the characteristic of each cell type is determined by the location of the neuron in the overall architecture, the strength of connection between cell types, and the type of connection, either inhibitory or excitatory. The individual cell groups were arranged according to Figure 1(a). The numbers of individual cells incorporated into our model, which was determined as a percentage of cells found in a single microzone [11], [12], [18], are as follows: mossy fibers −24, climbing fibers −3, granule cells −3300, Golgi cells −300, basket cells −130, Purkinje cells −15, and deep cerebellar neurons- 2. See Table 1 for the numbers used. Purkinje cells were arranged into three subgroups each containing 5 cells (see Figure 1(a)). Each subgroup received input from only one climbing fiber cell. Figure 1(b) depicts a snapshot from the simulation. The learning rate corresponds to how quickly a neuron adapts to training. It influences the importance of connection between particular cell types and thus can be correlated with the strength of that connection. The strength of the connection between climbing fibers and Purkinje cells was defined as approximately 26,000 times as strong as the rest of the connections in the cerebellum [7], [11], [24], with the exception of the connection between mossy fibers and granule cells, which was modeled to be four times as strong as the rest of the connections. In the model, both types of synaptic connections, namely excitatory and inhibitory, were incorporated. Only connections from Golgi cells to granule cells, basket cells to Purkinje cells, and Purkinje cells to deep cerebellar neurons are inhibitory, while the rest of the connections are excitatory. In our model of cerebellar microzone, as also found *in vivo*, mossy fibers and climbing fibers functioned as input cells, and deep cerebellar neurons were considered to be the output of the cerebellum.

**Table 1:** Comparison of the effect of cell death within each cell type.

Cell type	Total number of cells	Number of cells removed	Number of cells left	Resulting error
mossy fibers	24	8 (32%)	16	5.25%
climbing fibers	1	1 (33%)	2	6.94%
granule cells	3300	990 (30%)	2310	2.84%
granule cells (small)	330	99 (30%)	231	3.40%
Golgi cells	300	90 (30%)	210	4.91%
basket cells	130	29 (30%)	101	7.29%
Purkinje cells	15	5 (33%)	10	41.57%

Removal of 990 (or 30%) of granule cells resulted in the least performance degradation (2.63%) as compared to removal of 5 (or 32%) of Purkinje cells that resulted in the worst performance degradation (41.60%).

### Training

In order to reduce the simulation complexity and its runtime, the simulation was constructed so that inputs and outputs were in a simple numerical/vector form. The entire training set consisted of 140 samples and was constructed to resemble the eye puff and buzz conditioning training of a rabbit [11], [25], [26]. Table 2 lists an example of the training set. This particular problem provides a well-understood functionality of the cerebellum on which to base the network training and evaluation. Because information encoded by mossy fibers changes frequently but varies over a small range, the training set was constructed so that the numbers used for the mossy fibers part of the input vectors, bounded by 0 and 1, were evenly distributed in that range and changed slowly (variation between individual numbers is small, i.e. 0.906 and 0.916). Climbing fibers fire infrequently, however, their signals are strong and long lasting [18]. Therefore, their training values were encoded as either 0 or 1, and only one number was made to change between individual training samples, analogous to encoding by gray code (see Table 2, “Climbing fibers” column for an example). In the model, deep cerebellar neurons consisted of only two neurons. The set was constructed so that the neural output corresponded to the response of the rabbit during the training paradigm, where the rabbit learns to associate the eye puff with the ear buzz. The error was defined as a decrease in ability to associate the puff and buzz relation (see *Cerebellum Architecture Simulation* section for performance verification of the model). The training was terminated when the error (calculated to within 6 significant figures) reached 0%, which corresponded with the rabbit completely mastering the associated learning skill. The input to the network represents the electrical stimuli received by individual neurons. The output, however, corresponds to a specific skill learned. The error therefore corresponded to a divergence of performance from a trained puff/buzz association and was defined numerically (averaged sum squared error calculated over an epoch, where a single epoch contained all of the training sample patterns) and presented as a percentage of a loss of learned association.

**Table 2:** Example of a training sample used for the simulation.

mossy fibers					climbing fibers			deep cerebellar neurons	
MF_1	MF_2	MF_3	MF_4	MF_5	CF_1	CF_2	CF_3	DN_1	DN_2
0.96	0.24	0.83	0.57	0.47	0	0	0	1	0
0.55	0.93	0.59	0.08	0.01	0	0	1	1	0
0.14	0.35	0.55	0.05	0.34	0	1	0	1	0
0.26	0.25	0.29	0.78	0.79	0	1	1	1	0
0.84	0.62	0.76	0.93	0.31	1	0	0	0	1
0.25	0.47	0.75	0.13	0.53	1	0	1	0	1
0.81	0.35	0.38	0.57	0.17	1	1	0	0	1
0.54	0.01	0.37	0.52	0.92	1	1	1	0	1

Numbers are bounded in the [0,1] interval. Progression down the vertical columns corresponds to each iteration of training.

### Cerebellum Architecture Simulation

**Figure 2:**
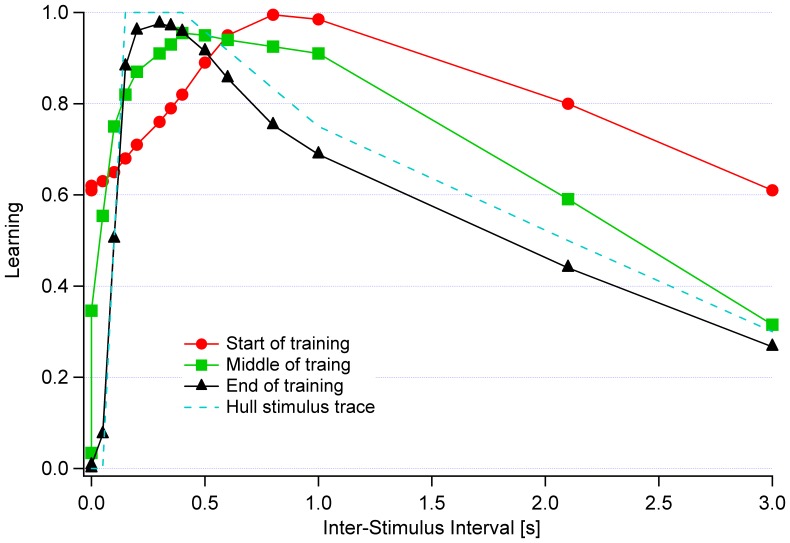
Comparison of performance of cerebellar microzone model when subjected to modulation of puff-buzz interval as compared to Hull's Stimulus Trace. The figure illustrates the change in performance throughout the training.

In order to substantiate the validity of our cerebellar microzone model, we subjected our model to Hull's test [11]. Hull's Stimulus Trace relates the ability to associate the auditory buzz and eye puff with an eye blink. In this test, an auditory buzz is followed by a puff to the rabbit's eye. The eye puff before the buzz is not associated with an eye blink; increasing the time lag of puff in relation to the buzz (beyond about 0.25 s) results in slowly decreased association. With the use of algorithm-specific variables, Emergent's error-driven learning (see section *Single Neuron Model*, and Equation 3) provides for time-shifting of various stimuli, such as eye puff and ear buzz, during training and testing phases. The validity of our model was verified by observing its output when the temporal separation of both stimuli was continuously increased (from simultaneous stimuli to an inter-stimulus interval of 3 seconds). Figure 2 demonstrates the performance of our model when compared to the Hull's stimulus trace. The figure portrays the behavior of the network throughout training, with three distinct phases: at the start of the training, in the middle of the training, and at the end of the training session. It can be seen that, in our model, as the training progresses the performance of the network becomes successively closer to the trace of the Hull's test, with the error for the start of the training, the middle of the training, and the end of the training being 34%, 16%, and 7%, respectively. This, we believe, substantiates the validity of our model.

### Procedure

The model consisted of seven cell types. Two of those types, climbing fibers and mossy fibers, were considered as inputs for the model. Deep cerebellar neurons were considered to function as the output (see Figure 1). This research concentrated on simulations of neurogenerative diseases that affect the intrinsic cell types of the cerebellum, namely basket cells, Golgi cells, granule cells, Purkinje cells, as well as mossy fibers and climbing fibers. Simulations of neurodegenerative diseases affecting individual cell types were conducted as well as a simulation of degeneration indiscriminately affecting all cell types.

**Figure 3:**
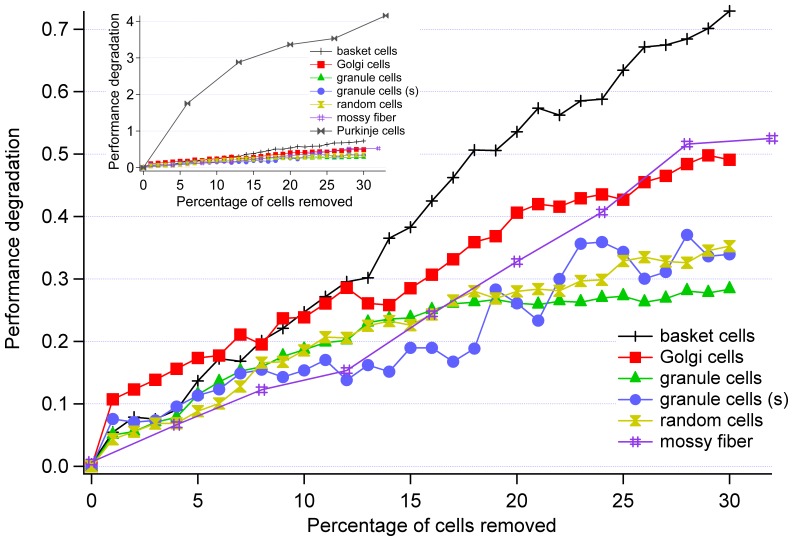
Performance degradation of cerebellum. Comparison of damage to individual cell types. Removal of Purkinje cells (included in the inset) results in the greatest performance degradation. Random cell removal corresponds to mossy fibers, basket, Golgi, granule, and Purkinje cells, with the likelihood of a particular cell types being damaged determined by the number of cells in relation to the overall number of cells in the model of cerebellum.

In the simulation, the network was trained using the aforementioned training set prior to simulating progressive cell death. Cell death was modeled by removal of a cell from the trained network (to simulate the progression of a disease affecting an adult animal, such as the rabbit that has undergone the puff/buzz conditional training). The progression of disease was modeled by removal of an increment of 1% of cells from an individual cell group starting at 0%, or no cell death, and continuing to 30% of cells removed. The exception to this was for the case of mossy fibers, which had one cell (4.0%) removed, climbing fibers, which had one cell (33.3%) removed, and Purkinje cells, which also had one cell (6.7%) removed. In these cases the simulation was terminated when 32%, 33.3%, and 33.3% of cells were removed, respectively. Figures 3(a) and 3(b) illustrate these procedures. Comparisons of performance degradation of individual cell types were made on equal percentage basis: 20% of Purkinje cells removed (or three cells) were compared to 20% of Golgi cells removed (or 26 cells) – see Figure 3 for graphical comparison. In the case of more generalized cell death that affects all cell groups (excluding mossy fibers, climbing fibers, and deep cerebellar neurons) in a random fashion, 1% of individual cell types were incrementally removed in a random fashion from different cell groups starting at 0% and terminating at 30%. For instance, removal of 1% of granule cells was followed by removal of 1% of basket cells. In the case of Purkinje cells, 1 cell was removed per simulation. In the generalized neurological model, the likelihood of a particular cell type being affected was determined by a probability based upon the ratio of the cell group to the total number of cells. For instance, the model consisted of 3300 *granule cells* and the total number of possible cells affected was 3745; hence, the likelihood of *granule cells* being removed was 88%.

**Figure 4:**
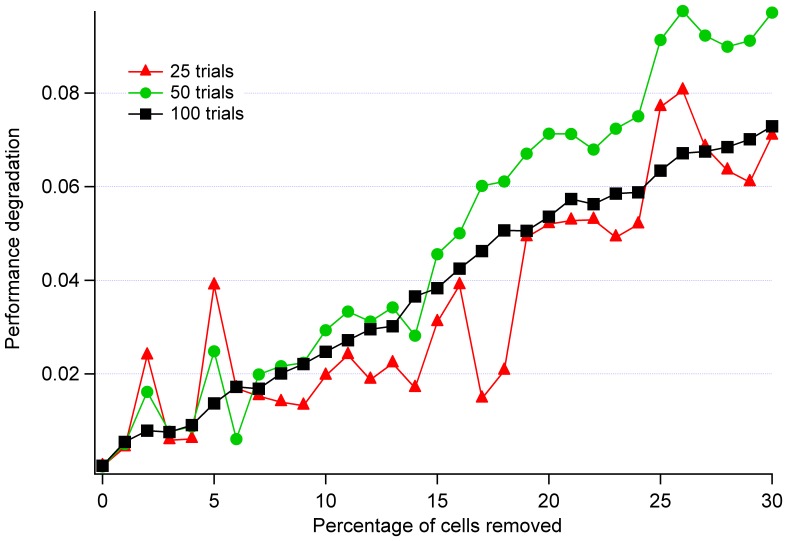
Performance degradation of cerebellum with basket cells removed after 25, 50, and 100 trials. Smoothness of the curve increases with the number of trials conducted (noise spikes are being removed).

Since individual trials frequently contained noise spikes, a total of 100 separate trials of each damage type were conducted and averaged in order to better elicit the functional form of the performance versus damage curves. Avoidance of local minima was verified by monitoring the training error graph and connection weights (local minimum occurs when no more weight adjustments is performed between training epochs [2], [27]). The smoothing effect of averaging can be seen in Figure 4. Performing 100 trials was found sufficient to obtain primarily monotonically increasing plots.

There is a wide body of evidence [28], [29] suggesting that, for a given size of training pattern, the number of neurons necessary to effectively encode the information has an optimal range: too few neurons cannot reasonably learn the necessary information, while too many neurons result in the network learning extraneous information. Roughly, the total number of cells in a network should equal the number of points in a training set (in our analysis a total number of cells was 3745, while the number of training points was 140×27 or 3780). Removing excessive neurons often results in increasing overall performance. For the purposes of our analysis we were concerned with an explicitly defined number of individual cell types. Hence, we decided that optimizing the number of individual cell types would invalidate the relationship between other cell types, and we did not perform any network size optimization. Additionally, analyzing graphs of individual cell death trials did not show significant variations (improvements of performance upon removal of neurons), which we interpreted as supporting that our training set was complex enough to deem most neurons necessary. For the purpose of this present work, removal of a given cell to simulate its death was permanent; no recovery or simulated neuroplasticity was implemented.

## Results

As discussed above, this model was used to study the functional effects of neurodegeneration within the cerebellum with the primary goal of understanding the importance of cellular organization on the loss of skills during the progression of a disease. In general, removal of cells from the simulation network, representing “cell death” in the circuit, resulted in a progressive increase of the error and hence loss of the trained association between auditory buzz and the air puff, as would be expected.

**Figure 5:**
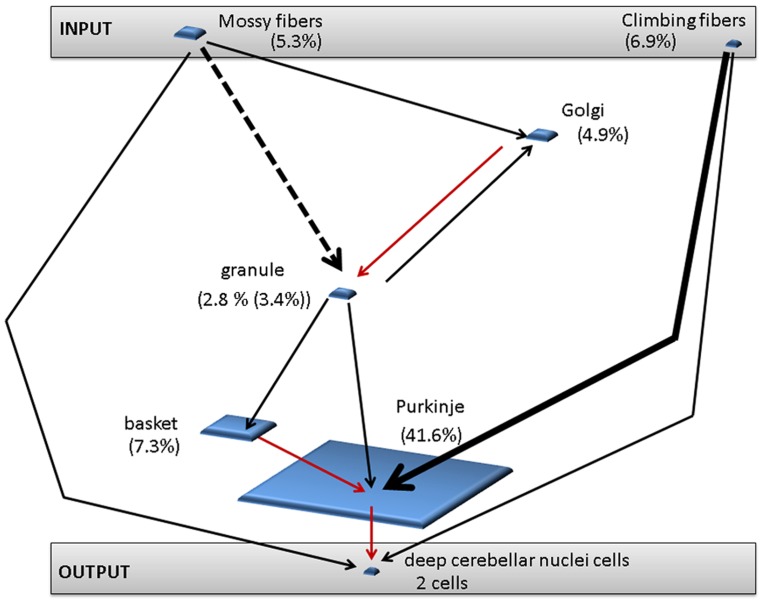
Comparison of a performance degradation of cerebellum vis-a-vis proximal relation of individual cell types to the deep cerebellar neurons. Numbers listed in parenthesis correspond to the performance degradation of individual cell types when maximum number of cells is removed. When the damage affects cell types the closest to cerebellar output (i.e. Purkinje cells), the performance degradation is significantly greater when the affected cells are located much further away (i.e. Golgi cells). For granule cells, the number in parenthesis represents the performance degradation when the total number of granule cells has been reduced by 90%.

**Figure 6:**
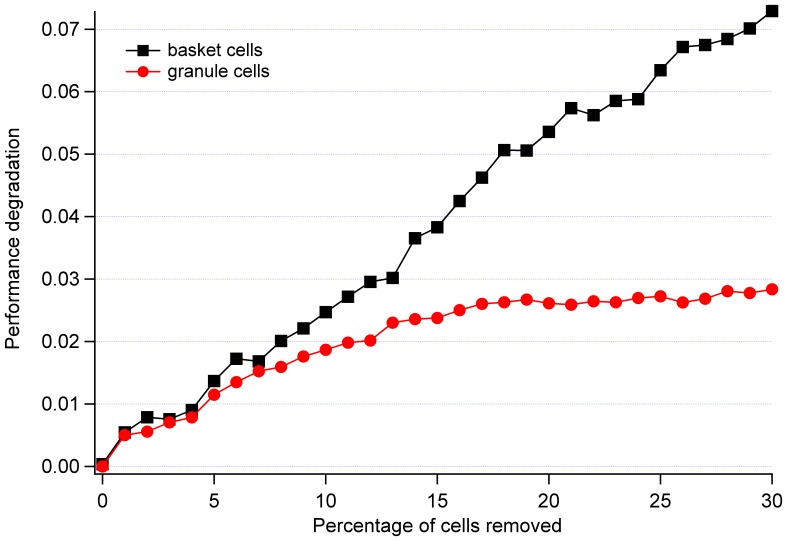
Comparison of a linear trend of basket cells and sub-linear trend of granule cells, when subjected to neural damage. It is believed that the differences are facilitated by the network topology and/or the existence of both excitatory and inhibitory connections in the model.

A major and unexpected finding of this study is a relationship between the proximity of the affected cells and the overall performance degradation of the entire circuit. As indicated in Figure 5 and Table 1, the closer the particular cell type is to the circuit output, the greater the functional deficit associated with cell death, compared on the same percentage basis. However, with the exception of climbing fibers, the cells that are further away from the output are also more plentiful than the cells located closer to the deep cerebellar neurons. Therefore, to elucidate the proximal relationship, we repeated the experiment with simulating death of granule cells. However, we reduced their total number by 90% to 330 cells. The result can be seen in Figure 6 and Table 1 labeled “Granule cells (s)”. The remarkable finding is that, even though the total number or granule cells was reduced to only 10% of the original size, the performance degradation of the entire network due to removal of granule cells still resembles that of the original network that contained significantly greater number of granule cells (roughly 3% degradation after removal of 30% of the cells). We believe that this is strong evidence that *the importance of cell types affected by cell death is very strongly related to the proximity of those cells to the output at the deep cerebellar neurons*. This finding is corroborated by Alstott *et al.* conclusion that the magnitude of the lesion effect depends on the lesion location [17]. From this we conclude that the intuitive notion that an “upstream” cell loss would result in greater damage than a “downstream” cell loss is incorrect. The explanation is as follows: the parallel and redundant nature of the network is able to some extent overcome neural damage further away from the circuit output (i.e. granule cells). However, the network has no mechanism for correcting the problem closer to the output (i.e. Purkinje cells); the altered data is more immediately sent to the circuit output and the circuit does not have enough time to fix the problem. In other words, when the damage is closest to the deep cerebellar neurons the percentage of information affected will be greater than if the damage happened further away. Cells that are adjacent to one another (i.e. Purkinje cells and deep cerebellar neurons) exert a greater influence over one another than cells separated by another cell (i.e. basket cells and deep cerebellar neurons).

We also investigated the hypothesis that the high performance degradation due to lesion of Purkinje cells was more influenced by their relative smaller number coupled with the information convergence rather than their proximity to the cerebellar output. We conducted two series of experiments where the information convergence was occurring at different proximity to the model’s output (data not shown). In most of the cases, it was the location of the affected cells with respect to the output that indicated the gravity due to cell loss and not the location of the neural convergence. This, we believe, further supports our hypothesis regarding cell location being the main indicator of the gravity of the functional deficit due to cell loss.

An additional finding of interest is that the performance degradation of some cell types (mossy fibers, Golgi cells, and basket cells) exhibit a linear trend when subjected to damage, while others (granule cells and Purkinje cells) display sublinear behavior, which can be fit reasonably well by a logarithmic function seen in Figure 6. We were not able to identify a particular factor (i.e., linear progression being associated with excitatory or inhibitory connections) that would explain this finding. However, we suggest that this is a consequence of the particular network topology and distribution of both excitatory and inhibitory connections considered.

Analysis of the simulation of a more generalized neurodegeneration or cell death in which cells were removed at random needs to consider that cell removal was influenced by the relative likelihood of cells of a particular type (from a particular layer in a computational model) being removed. The probability of removing a granule cell from the overall network was about 88% while the likelihood of removing a Purkinje cell was 0.4%. Therefore, the performance degradation due to removal of random cells is more similar to that of granule cells than that of other cells. However, it should be noted that this behavior is to be expected in biological networks, namely a disease that less discriminately affects cell types is more likely to affect cells that are more plentiful.

The last issue that needs to be addressed is that of inhibitory connections. In a simulation environment, the output of a neuron is computed by summing all of the input signals. The input from the excitatory connection is positive while the input from the inhibitory connection is negative. Removal of an inhibitory connection, or individual cells from an inhibitory connection type (layer), usually results in the numerical increase of the output, while the removal of excitatory neurons is followed by the numerical decrease of the output. Nevertheless, because it has the immediate effect of modifying the computed (during training) outcome, the cell death of inhibitory neurons results in error increases similar to those due to cell death of excitatory neurons. In biological systems, an excessive loss of inhibition could lead to excitotoxicity in the downstream neuron (and additionally lead to cell death of excitatory neurons).

## Conclusions

This computational analysis focused on the effects of neurodegeneration of cerebellar neurons in patterns consistent with different types of neurological diseases. The most significant result was that the overall performance of the cerebellum may not be as strongly influenced when a disease affects cells that are relatively plentiful or that are more separated from the output of the cerebellum. This indicates that with diseases that affect specific cell types, the overall consequence on the affected animal could be predicted in part based upon the relative proximity of targeted cells to other cells that are implicated in specific functions.

Finally, the present research concentrated on the performance degradation and functional effects of cell death within a well-defined neuronal circuit. We suggest that it provides an early and clear example of using computational analysis to study the effects of biologically realistic neuronal network degradation. We note that the simulation was done to mimic the architecture of the cerebellum on a very gross scale. Details about physiological differences between neurons in the different sub-circuits were not included here. However, we do not expect such differences to qualitatively change the results, since we modeled damage corresponding to complete removal of individual neurons. In the future, modeling of other types of neurological disease would provide insight into performance degradation and the pathological consequences of disease not resulting from cell death, but caused by alterations of cellular excitability and/or firing patterns. Such a change in cellular behavior can produce a cascade of effects on a neuronal circuit and ultimately on nervous system function. We further suggest that such computational analysis of specific sub-circuits of the brain can provide progress toward a complete understanding of the functional consequences of different patterns of neurodegeneration and for assessing effective cellular targets for potential treatments of human ataxias and neurodegenerative diseases.
